# Ceftriaxone-resistant *Salmonella* Typhi Outbreak in Hyderabad City of Sindh, Pakistan: High Time for the Introduction of Typhoid Conjugate Vaccine

**DOI:** 10.1093/cid/ciy877

**Published:** 2019-02-15

**Authors:** Mohammad Tahir Yousafzai, Farah Naz Qamar, Sadia Shakoor, Khalid Saleem, Heeramani Lohana, Sultan Karim, Aneeta Hotwani, Shahida Qureshi, Naveed Masood, Mudasir Rauf, Jamshed Ahmed Khanzada, Momin Kazi, Rumina Hasan

**Affiliations:** 1 Department of Paediatrics and Child Health, Karachi; 2 Department of Pathology and Laboratory Medicine, Aga Khan University, Karachi; 3 Department of Paediatrics and Child Health, Aga Khan Maternal and Child Center, Hyderabad; 4 Provincial Disease Surveillance Unit, Director-General Health Office, Pakistan; 5 District Polio Control Room, Hyderabad, Sindh, Pakistan

**Keywords:** ceftriaxone resistance, typhoid, TCV, outbreak, XDR

## Abstract

**Background:**

The Aga Khan University clinical microbiology laboratory identified an outbreak of ceftriaxone-resistant *Salmonella* Typhi in Hyderabad, Pakistan, through antimicrobial resistance surveillance. An outbreak investigation was carried out to identify the risk factors and institute control measures. Here we report the preliminary findings of this outbreak investigation, using data collected from 30 November 2016 to 28 March 2017.

**Methods:**

The design for the investigation was a case-control study that included identification of culture-proven ceftriaxone-resistant *S*. Typhi cases, suspected cases from the households or neighborhood of the confirmed cases, and enrollment of controls matched by age to identify the risk factors. Data were collected through face-to-face interviews using a structured questionnaire. Blood cultures were obtained from all suspected cases. Drinking water samples from each household of cases and controls were obtained for microbiological testing. Geographic Information System coordinates were obtained for all cases and controls.

**Results:**

Only 2 subdistricts of Hyderabad (Latifabad and Qasimabad) were affected. A total of 101 confirmed cases of ceftriaxone-resistant *S*. Typhi had been reported in 4 months with the first case reported on 30 November 2016. Median age was 48 (interquartile range, 29–84) months. The majority (60% [61/101]) of the cases were 6–60 months old. More than half (56% [57/101]) of the cases were male. About 60% of the cases were admitted to hospital and treated as inpatient. More than half (57/101) of the patients developed complications related to typhoid.

**Conclusions:**

Community awareness was raised regarding chlorination of drinking water and sanitation measures in Hyderabad. These efforts were coordinated with the municipal water and sewage authority established to improve chlorination at processing plants and operationalize fecal sludge treatment plants. Outbreak investigation and control efforts have continued. Immunization of children with typhoid conjugate vaccine within Hyderabad city is planned.

Typhoid is endemic in South Asia and is the most common bacteremic illness in children in Pakistan, with rates as high as 1000 cases per 100 000 child-years having been reported from Karachi [1]. Increasing antimicrobial resistance in *Salmonella* Typhi is a critical challenge. Globally, multidrug resistance (MDR) and fluoroquinolone resistance is increasingly reported in *Salmonella enterica* subspecies *enterica* serovars Typhi and Paratyphi from Asia and Africa [2]. The emergence of MDR, especially fluoroquinolone resistance, has severely limited therapeutic options in high-disease-burden countries such as Pakistan. The first report of ceftriaxone resistance in *S*. Typhi was from Bangladesh in 1999 [3]. Ceftriaxone resistance has remained rare, with <1% of strains resistant during 2009–2014 in Pakistan [4, 5], and ceftriaxone continues to be the first therapeutic option for the inpatient management of typhoid. However, due to the ever-present threat of emerging resistance, the World Health Organization recommends ongoing surveillance for antimicrobial resistance to identify and address any change in resistance in a timely manner [6].

Typhoid is a notifiable disease in Pakistan, and the Aga Khan University (AKU) Hospital reports blood culture–confirmed and suspected cases of enteric fever to the provincial health department. Laboratory surveillance at the AKU Hospital detected an outbreak of ceftriaxone-resistant typhoid fever due to *S*. Typhi in November 2016 from the city of Hyderabad of Sindh, Pakistan. All culture-confirmed cases were confirmed to be from Hyderabad, and identification of blood culture isolates and their susceptibilities were reconfirmed by routine biochemical and minimum inhibitory concentration (MIC) methods, respectively. Once confirmed, the provincial health department was alerted and an outbreak investigation was initiated.

As of the end of March 2017, 101 cases of ceftriaxone-resistant *S*. Typhi have been identified. This is the largest outbreak of ceftriaxone-resistant *S*. Typhi reported in the world. Here we present a brief report of this outbreak, the epidemiology, and the geospatial mapping of the cases.

The outbreak investigation is ongoing and final results of the outbreak investigation and the case-control study will be reported after data analysis. The purpose of this early report is to update the scientific community on ongoing investigations and efforts.

## METHODS

### Study Settings

Hyderabad is the second-largest city of Sindh Province, located in the southeast of Pakistan. Hyderabad consists of 4 *talukas* (subdistricts), named Hyderabad urban, Hyderabad rural, Qasimabad, and Latifabad. The total population of Hyderabad is approximately 1.8 million, with the distribution across the 4 talukas as follows: Hyderabad urban (population 564 380), Hyderabad rural (population 450 235), Qasimabad (population 300 609), and Latifabad (population 577 809). The ceftriaxone-resistant *S*. Typhi outbreak predominantly occurred in Qasimabad and Latifabad areas. Three large hospitals serve the population of Latifabad and Qasimabad. These are (1) the Aga Khan Maternal and Child Center (AKMCC), a subsidiary of AKU Hospital serving only women and children; (2) the civil hospital Hyderabad, a large tertiary-care public-sector teaching hospital providing free of cost diagnostic and treatment facilities; and (3) Bhittai hospital Hyderabad, a tertiary-care public-sector hospital providing diagnostic and treatment facilities. In addition, there are several other private clinics and smaller hospitals such as Taluka Hospital Qasimabad and Taluka Hospital Latifabad located in the target areas of Hyderabad.

The AKU, the Centers for Disease Control and Prevention Field Epidemiology and Lab Training Program, and the health department of Hyderabad worked together to establish sentinel surveillance sites at AKMCC and Taluka Hospital Qasimabad, to identify suspected cases of ceftriaxone-resistant *S*. Typhi.

### Study Design

The outbreak investigation is a case-control study and activities were based on identification of culture-proven, ceftriaxone-resistant *S*. Typhi cases from the laboratory surveillance of the AKU clinical microbiology laboratory (AKU has several satellite laboratory collection points in Hyderabad), sentinel hospitals, and suspected cases from households or neighborhood of the confirmed cases and enrollment of 4 controls matched by age for each confirmed case to identify the possible source of this outbreak.

A confirmed case was defined as having ceftriaxone-resistant *S*. Typhi on blood culture; a suspected case was any subject with fever for at least 3 days during the last 7 days in the absence of any other focus of infection and living within the household or neighborhood of a confirmed ceftriaxone-resistant typhoid case. Controls were healthy subjects, without any history of illness during the last 4 weeks, matched by age to the cases by ±1 year. The team conducted household visits of each confirmed case and their corresponding matched controls. Interviews using the structured outbreak investigation questionnaire were conducted with each case and control in the presence of their parents or guardians. Geographic Information System coordinates were also marked for both case and controls.

All blood samples from AKMCC were tested at the AKU clinical microbiology laboratory; blood samples from Qasimabad hospital and suspected cases in the community were tested in the infectious disease research laboratory (IDRL) of AKU, Karachi. Blood samples were stored at room temperature (22°C–28°C) and transported to the IDRL Matiari branch (30 km from Hyderabad) within 8 hours of sample collection. Both the AKU clinical microbiology laboratory and the IDRL used similar methods for blood culture (BACTEC 9240 automated system, Becton Dickinson). Methods for isolate confirmation and susceptibility testing and confirmation are described in previously published literature [7]. Water samples from households of both cases and controls were tested microbiologically for presence of fecal coliforms and *Escherichia coli* (as indicators for fecal contamination) and *Salmonella* species at the AKU clinical microbiology laboratory by the membrane filtration method [8, 9]. In brief, 100 mL of water was filtered through Millipore membrane (Merck) and cultured for coliforms and thermotolerant *E. coli*.

Expedited ethical approval was obtained from the ethical review committee of AKU.

## RESULTS

### Preliminary Findings

A total of 101 cases were detected during 30 November 2016 to 28 March 2017 from the AKU clinical microbiology laboratory alone. All of the cultured isolates were resistant to ampicillin, chloramphenicol, cotrimoxazole, and ciprofloxacin, and exhibited ceftriaxone MICs of >64 µg/mL but remained sensitive to azithromycin, imipenem, and meropenem. All the isolates of *S.* Typhi were extended-spectrum β-lactamase (ESBL) producing.

**Figure 1.**
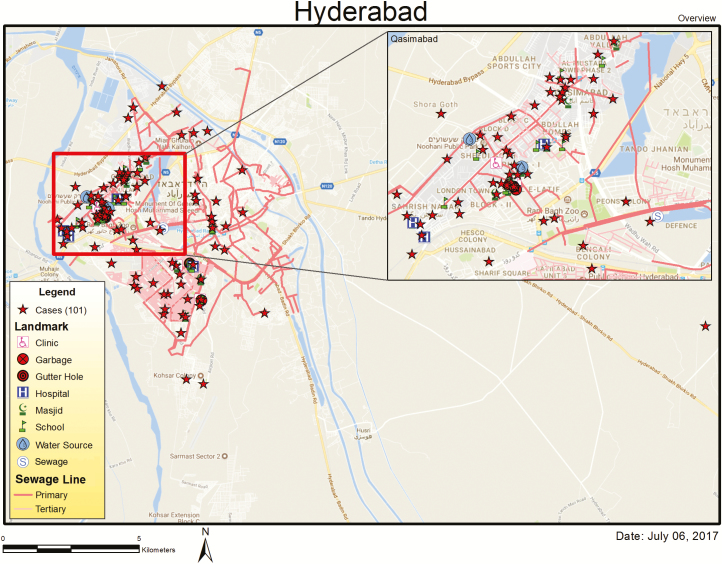
Geospatial map of ceftriaxone-resistant cases (N = 101).

Geographically, all of these cases belonged to the 2 adjacent talukas (Latifabad and Qasimabad) of Hyderabad. The geospatial map of the 101 cases revealed the clustering of cases in Latifabad and Qasimabad talukas of Hyderabad (Figure 1). Slightly more than half of the cases (57/101 [56.4%]) were male. Median age of the patients was 48 (interquartile range, 29–84) months. Only 5 cases were aged 6–12 months, whereas more than half (56/101 [55.4%]) of the cases were 2–5 years of age. About 60% of the patients (61/101) needed hospitalization for the treatment while the remaining 40% were treated on an outpatient basis (Table 1).

**Table 1.** Age and Sex Distribution of Ceftriaxone-resistant *Salmonella* Typhi Cases (N = 101) in Hyderabad, Pakistan, November 2016–March 2017

Characteristic	No. (%)
Sex	
Male	57 (56.4)
Female	44 (43.6)
Age, mo, median (IQR)	48 (29–84)
Age group, y	
≤1	5 (5.0)
2–5	56 (55.4)
6–10	27 (26.7)
11–15	4 (4.0)
16–20	5 (5.0)
≥21	4 (4.0)
Hospitalization	
Yes	61 (60.0)
No	40 (40.0)
Clinical characteristics	
Fever	101 (100)
Duration of fever, d, median (range)	14 (2–69)
Cough	28 (27.8)
Difficulty breathing	12 (11.9)
Abdominal pain	44 (43.6)
≥3 watery stools/d	32 (31.7)
Constipation	8 (7.9)
Vomiting	52 (51.5)
Visible blood in stool	2 (2.0)
Jaundice	5 (5.0)
Headache	35 (34.6)
Seizures	6 (5.9)
Confusion	2 (2.0)
Rash	2 (2.0)
Complications	
Free peritoneal fluid	51 (50.5)
Gastrointestinal bleeding	2 (0.2)
Intestinal perforation	2 (0.2)
Encephalopathy	1 (0.9)
Hepatitis	1 (0.9)
Outcome (death)	1 (0.9)

Abbreviation: IQR, interquartile range.

All of the cases developed fever and the median duration of fever was 14 (range, 2–69) days. Vomiting was the most common symptom (52/101), followed by abdominal pain (44/101). More than half of the patients (57/101) developed some kind of complication including free peritoneal fluid (n = 51), gastrointestinal bleeding (n = 2), intestinal perforation (n = 2), encephalopathy (n = 1), and hepatitis (n = 1). Cases were successfully treated with either azithromycin or a carbapenem (meropenem or imipenem). There was 1 death among the patients (Table 1).

**Figure 2.**
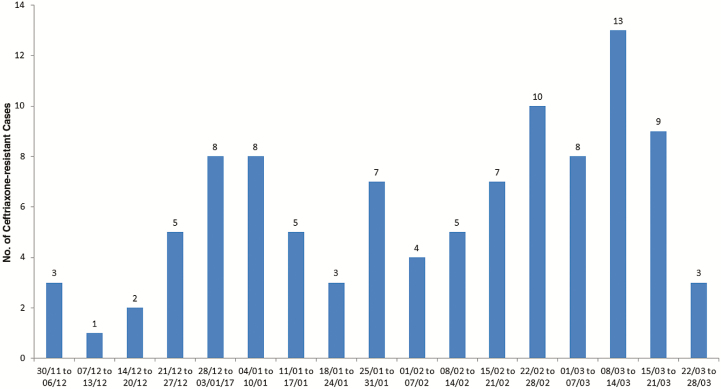
Weekly distribution of ceftriaxone-resistant *Salmonella* Typhi cases (N = 101) during 30 November 2016 to 28 March 2017 in Hyderabad, Pakistan. Dates are presented as day/month.

The number of weekly reported cases varied from as low as 1 during the beginning of the epidemic to as high as 13 during the week of 8–14 March 2017. The epidemic curve showed a cyclical trend that shows propagated transmission of the ceftriaxone-resistant *S*. Typhi (Figure 2) The culture result of 60 household drinking water samples (not treated at the household level) revealed very high counts for thermotolerant *E. coli*, but no samples were positive for *Salmonella*. Presence of high (>1 colony-forming unit/100 mL of water) amounts of *E. coli* indicated fecal contamination of potable water in Hyderabad.

## DISCUSSION

### A Costly Emerging Public Health Problem

To date, this is the largest outbreak of ceftriaxone-resistant *S*. Typhi to have been reported. This is an emerging public health problem in Pakistan, as the retrospective surveillance data from the AKU clinical microbiology laboratory showed only 4 cases (0.01%) of ceftriaxone-resistant *S*. Typhi from Pakistan during the last 3 years (2012–2015) [5]. Another study from Karachi, Pakistan, also reported <1% ceftriaxone-resistant *S*. Typhi during 2009–2012 [4]. However, >100 culture-confirmed cases observed in 4 months that constituted this outbreak from 2 subdistricts demonstrate the magnitude of ceftriaxone-resistant *S.* Typhi in Pakistan. Whole-genome sequencing of the isolates confirmed a novel strain of *S.* Typhi belonging to the H58 lineage harboring plasmid encoding additional resistance elements, including the *bla*_CTX-M-15_ ESBL, and carrying the *qnrS* fluoroquinolone resistance gene [7]. A recent study by Klein et al [10] globally compared the increase in consumption of antibiotics from 2000 to 2015 and reported that Pakistan faced a 65% increase in defined daily doses of antibiotics (increase from 1.3 million doses in 2000 to 800 million doses in 2015). Moreover, the majority of the antibiotic use is empirical, easily available over the counter, and not clinically justified owing to the lack of any health regulation across the country. Overuse of antibiotics can be an important driver of emerging widespread drug-resistant pathogens including *S.* Typhi.

This new public health problem has repercussions for the health system, including an increase in the cost of treatment. As the outbreak strain was MDR, including resistant to fluoroquinolones, cases were treated with azithromycin and a carbapenem alone or in combination, with average cost of treatment increasing by US$2 per dose per day. In addition, most cases of typhoid are diagnosed clinically or through serology and treated in the communities without a blood culture and sensitivity; therefore, most patients could not receive timely doses of appropriate antibiotics, resulting in prolonged illness and higher number of complications. This demonstrates that the outbreak has had a significant impact on the costs of treatment of enteric fever in Hyderabad. If not controlled quickly, the outbreak would significantly burden the fragile healthcare system in Pakistan and increase patients’ out-of-pocket health expenditures.

### Identifying Interventions

The weekly distribution of cases since the reporting of the first case on 30 November 2017 revealed an irregular pattern with at least 2 peaks during the 4-month duration of this outbreak, suggesting a common source epidemic. Although the case-control study is not yet concluded to elucidate the possible cause of this outbreak, geospatial mapping revealed clustering of the cases around sewage lines. Furthermore, field visits by AKU staff and meetings with the water and sewage authority (WASA) of Hyderabad identified several shortcomings in the existing water distribution and sewage treatment system. There are 4 sewage sludge treatment plants in Hyderabad, but none of them have functioned for the past year. All the sewage goes directly into the river. The water distribution lines are old and seepage is common from these lines; vacuum pumps are used in the periods of low or no municipal water supply and enhance the contamination of water from damaged pipes. Therefore, the probability of contaminated water as a potential source of ceftriaxone-resistant *S.* Typhi is high. Household water cultures conducted so far had a high *E. coli* count indicating sewage contamination, but none were positive for *S*. Typhi. Samples have been saved for *S.* Typhi for polymerase chain reaction as it is reportedly a more sensitive test for the identification of *S.* Typhi in water [11]. The outbreak has continued, and cases are consistently occurring every week, further demonstrating that the existing control interventions without mass immunization are not successful in breaking the chain of transmission.

Generally, typhoid is considered a disease of young adults, with higher incidence among the age group ≥15 years [4]. In Pakistan, previous studies have shown lower incidence of sensitive *S*. Typhi among young children compared with young adults [4]. In this outbreak, 60% of cases were <5 years of age, and this has implications for the control of the outbreak. The polysaccharide vaccine (Typhim Vi) manufactured by Sanofi Pasteur (Lyon, France) [12], which is licensed and available on the private market in Pakistan, has poor immunogenicity among children <2 years of age. Therefore, this vaccine was not considered a useful containment strategy for this outbreak, which predominantly affected infants and children <2 years of age. In addition, the polysaccharide vaccine confers protection for only 3 years, resulting in repeated booster dose requirement every 3 years [13]. The new-generation typhoid conjugate vaccine (Typbar-TCV, Baharat Biotech International, India [TCV]) has demonstrated better safety, immunogenicity, and longer protection compared with polysaccharide vaccine across all age groups, including infants, making it a better option for this outbreak [14]. As Typbar-TCV was not licensed in Pakistan, special permission from the drug regulatory authority of Pakistan was therefore needed to import TCV for this outbreak.

### Interventions

Both vaccination and efforts to improve water, sanitation, and hygiene are being undertaken to combat this outbreak. Community awareness and education about the outbreak and prevention strategies have started. This includes education around hygiene, sanitation, boiling of drinking water, thorough washing of raw vegetables and fruits, and the risks of eating from street vendors. Awareness sessions for general physicians and pediatricians to play a positive role in community awareness and outbreak control have also been conducted. In addition, meetings with the higher authorities of WASA regarding the operationalization of fecal sludge treatment plants have been conducted. Technical and operational barriers have been identified during these meetings, and efforts are under way to overcome these barriers and to operationalize the sludge treatment plants as soon as possible. WASA has been requested to ensure an uninterrupted supply of water to the affected areas.

AKU considered multiple immunization strategies to control the outbreak. Mass immunization of children aged 6 months–5 years with a single dose of TCV would cover only 60% of the population at risk and help contain the outbreak. On the other hand, targeting an age group of 6 months–10 years would cover 87% of the population at risk, resulting in broader coverage and better protection against the spread of this outbreak. Therefore, it was decided to target children 6 months–10 years of age, and AKU obtained special approval for import and use of TCV in Hyderabad for containment of the outbreak. Piloting of the mass vaccination was started in last week of January 2018.

We could not establish sentinel surveillance at all of the hospitals and first level care facilities (primary care facilities) within the target community. Physicians are treating cases of fever empirically with antibiotics, which indicates that the cases reported here are just the tip of the iceberg.

With current ongoing surveillance, investigations, and containment efforts, there is hope that the outbreak can be contained. The information being collected is vital to combating not only this outbreak, but setting precedence for elimination efforts for MDR typhoid in the country and region.

## Notes

***Disclaimer.*** The granting agency had no role in the writing or analysis of the manuscript.

***Financial support.*** F. N. Q. has received research training support from the National Institute of Health Fogarty International Center (grant number 1 D43 TW007585-01).

***Supplement sponsorship.*** This supplement was sponsored by Coalition against Typhoid, Sabin Vaccine Institute, Bill & Melinda Gates Foundation.

***Potential conflicts of interest.*** All authors: No reported conflicts. All authors have submitted the ICMJE Form for Disclosure of Potential Conflicts of Interest. Conflicts that the editors consider relevant to the content of the manuscript have been disclosed.
